# Legacy-Oriented Interventions for Supporting Family Bereavement and Psychosocial Adaptation in Pediatric Palliative Care: A Systematic Review

**DOI:** 10.1177/10499091251383152

**Published:** 2025-09-24

**Authors:** Daniela Jesus-Ferreira, Paulo Reis-Pina

**Affiliations:** 1 Palliative Medicine Center, Faculty of Medicine, University of Lisbon, Lisboa, Portugal; 2 Acute Palliative Care Inpatient Unit, Local Health Unit of Santa Maria, Lisboa, Portugal

**Keywords:** bereavement, family, palliative care, parent-child relations, pediatrics, psychosocial support systems, systematic review, terminal care

## Abstract

**Background:**

Legacy-oriented interventions (LOI) are increasingly integrated into pediatric palliative care (PPC) to support children and their families during serious illness and bereavement. However, its impact on family grief and psychosocial outcomes remains insufficiently explored.

**Objectives:**

This systematic review evaluated the effects of LOI on children and families receiving PPC.

**Methods:**

A systematic search was conducted in PubMed, Scopus, and Web of Science up to February 28, 2025. Eligible studies included children and families in PPC settings. Outcomes of interest encompassed relational and memorialization, psychosocial adaptation, family/care system, and clinical domains. Quality appraisal and narrative synthesis were performed.

**Results:**

Nine qualitative studies met inclusion criteria, all conducted in the United States of America with 164 participants. Eight studies were rated as high quality and one as moderate. LOI facilitated emotional connection with the child, supported coping and meaning-making, and fostered sustained relationships with healthcare professionals. Several interventions were adapted to cultural or spiritual contexts. The effectiveness of LOI varied by type of legacy (tangible vs intangible) and the family’s stage of bereavement. Few studies explored symptom control or broader social outcomes.

**Conclusions:**

LOI appear to provide valuable psychosocial support for families in PPC, particularly by sustaining emotional bonds and promoting grief adaptation. Nevertheless, the evidence base is limited to small, qualitative studies from a single country. Further research in diverse cultural settings, employing longitudinal and mixed-method designs, is needed to inform clinical practice and policy.

**What Is Already Known About the Topic**

• Legacy-oriented interventions are used in pediatric palliative care to support emotional well-being.
• Their potential impact on grief and other psychosocial outcomes remains underexplored.

**What This Paper Adds**

• This systematic review identifies key psychosocial outcomes including bond maintenance, coping, meaning-making, and family engagement.
• It underscores the need to personalize interventions according to family values, grief stages, and cultural context.
• Few studies explore symptom control or the broader social impact of legacy-oriented interventions.

**Implications for Practice, Theory or Policy**

• Legacy-oriented interventions should be integrated into comprehensive psychosocial care in pediatric palliative care.
• Healthcare teams should actively involve families in designing interventions aligned with their needs and values.
• Further research is needed to inform culturally relevant and scalable legacy-oriented intervention practices.

## Introduction

### Rationale

The death of a child has profound consequences for families, disrupting daily functioning and affecting physical, psychological, and relational well-being.^1^ Parents who lose a child are at heightened risk of prolonged grief due to the intensity and persistence of their symptoms.^2^

Children with serious health conditions may benefit from pediatric palliative care (PPC), which provides holistic support that addresses medical, psychosocial, and spiritual needs.^3^ Within PPC, interventions are encouraged that prioritize the child’s best interests while also supporting families.^3^

In palliative medicine, legacy-oriented interventions (LOI) have become increasingly common.^2^ LOI are structured activities conducted within PPC to support children and families in creating enduring memories and symbolic representations of their lives, values, and relationships.^4-8^ Legacy activities are usually co-created by children, families, and healthcare professionals, with the dual aim of producing keepsakes and fostering meaningful experiences during illness and bereavement.^9-11^

Legacy activities may take tangible forms, such as photography, songwriting, letter writing, memory books, artwork, or handprints,^2,4,12,13^ or intangible forms, such as inspiring others, continuing family rituals, donating belongings, or leaving an emotional impact on parents, siblings, and the wider community.^5-7,11,14-18^ Both forms emphasize relational continuity and provide opportunities for meaning-making and emotional connection that extend beyond the child’s physical presence.^6,7,15^

The essential elements of LOI are: (i) *Intentionality* – explicitly preserving the child’s memory and identity for the family and future generations^5,8^; (ii) *Participation* – active engagement of the child, family, and sometimes healthcare professionals^4,7^; (iii) *Continuity* – providing a lasting representation that fosters bonds, meaning-making, and coping during illness and after death^6,11^; and (iv) *Personalization* – tailoring to the child’s identity, values, cultural background, and preferences.^18,19^

The significance of LOI lies in their ability to reflect the individuality of the child within their relational context.^18,19^ They differ from general supportive care by their specific focus on creating tangible or symbolic legacies that extend beyond the illness trajectory and contribute to healthier bereavement adaptation.^6,7^ Evidence suggests that LOI not only provide lasting reminders of the child’s life but also foster meaningful shared experiences and support the development of coping strategies for families.^4,9-11,20^

Despite increasing use in PPC, the long-term effects of LOI on bereavement remain underexplored, particularly compared to adult populations.^2,4,9^ Preliminary studies suggest that legacy projects may aid coping and adaptation in bereavement,^2,10^ but a comprehensive synthesis of this evidence is lacking.

### Objectives

This systematic review aimed to evaluate the impact of LOI in PPC, with particular emphasis on their role in supporting children and families during end-of-life and bereavement.

The review was guided by the research question: How do LOI support family bereavement and psychosocial adaptation in PPC?

## Methods

This systematic review followed the recommendations of the “Cochrane Handbook for Systematic Reviews of Interventions”,^21^ and was reported in accordance with the Preferred Reporting Items for Systematic Reviews and Meta-Analyses guidelines.^22^

### Eligibility Criteria

Studies were selected based on the following criteria.

• **Participants:** Pediatric population and their family members or bereaved parents in palliative care or end-of-life care.
• **Interventions:** LOI.
• **Comparator/control:** usual or standard care, if present.
• **Outcomes:** Relational and memorialization, psychosocial adaptation, family/care system, or clinical domains relevant to children and families in PPC.
• **Study designs:** All study types were considered.

### Information Sources

The PubMed, Scopus, and Web of Science databases were searched. The database search was conducted in February 28, 2025.

### Search Strategy

The search included both free text terms and MeSH terms. The structure was as follows: ((“palliative care”) OR (“hospice care”) OR (“terminal care”) OR (“end of life”)) AND ((pediatr*) OR (NICU) OR (neonatal) OR (“new born”)) AND legacy AND (bereav* OR Grief OR griev*). No filters were applied.

### Selection Process

Both authors independently screened all articles by title and abstract. The full text of relevant articles was then assessed for eligibility, also independently by both authors. Any inconsistencies or discrepancies in study selection were resolved through discussion, with a consensus reached between the authors. No automation tools were used in this process.

### Data Collecting Process

A data-charting form was created using Microsoft Excel (Microsoft Corp, 2016) and iteratively refined through testing. The first author primarily conducted data charting, with the second author independently cross-verifying and augmenting the data. Any discrepancies between reviewers were resolved through discussion until consensus was achieved. Original authors were not contacted for additional data or confirmation. No automation tools were employed in this process.

### Data Items

Data were extracted on relational and memorialization, psychosocial adaptation, family/care system, or clinical domains relevant to children and families in PPC.

Additionally, information was extracted on several other variables, including authors, country of origin, year of publication, study design, population characteristics, study objectives, interventions, key observations, and main findings.

### Study Risk of Bias Assessment

All the included studies were subjected to rigorous appraisal by two independent reviewers. Any disagreements were resolved through team discussion until consensus. The Joanna Briggs Institute critical appraisal checklist for qualitative research was used.^23^ A decision was made for each study to either include, exclude, or seek additional information. Study quality was assessed using the proportion of “Yes” items to the total number of items. Studies were classified as low quality if they scored below 0.5, moderate quality if they scored between 0.5 and 0.7, and high quality if they scored above 0.7.

### Effect Measures

The effect measures reported by the authors were accepted and used in both the synthesis and presentation of the results.

### Synthesis Methods

The evidence is presented in a narrative format. To summarize our findings, we prepared three tables: Table 1 outlines the characteristics of the included studies, Table 2 presents the main findings, and Table 3 provides a critical appraisal of the evidence.

**Table 1.** Characteristics of the Included Studies (n = 9)

Study Country, year	Study design	Objectives	Population	Method	Intervention	Observations
Barta et al, USA, 2024^11^	Qualitative study	To evaluate parents’ preferences and needs for grieving support as a reference for best practice in LOI.	-6 bereaved parents who are also mentors (83% mothers; aged 42–62 years) Decedents’ age: from 9 months to 25 years	Semi-structured interviews	Two focus groups were held to interview bereaved parents about their perspectives on the LOI provided to their children and their end-of-life experiences (eg, types of activities, emotions, connections, timing, assessment)	-The participants also work as volunteers in a hospital, mentoring other parents whose children also receive LOI. -Their judgement is based on their experience and that of the other parents
Andrews et al, USA, 2020^12^	Qualitative study	To determine the effect on bereaved parents of MTHS, as a legacy-making	-12 bereaved parents (83% mothers) -Decedents’ age: from 8 days to 16 years	Online survey, with subsequent follow-up calls	-A digital stethoscope was used to record the child’s heartbeat and then create a phonocardiogram. This intervention was included in the “Music -Therapy Heart Sounds” programme with the aim of creating a digital keepsake. -The importance of this LOI, its use after death and recommendation to other parents were explored	This study includes parents’ whose children died from both oncologic and non-oncologic diseases
Ramirez et al, USA, 2019^13^	Qualitative study	To explore the importance of professional photography as a legacy to support parents grieve	- 23 participants: 6 bereaved parents (66.6% mothers); 8 photographers; and 9 health care professionals (33.3% nurses, 22.2% social workers, 11.1% pediatricians, 11.1% chaplain, 11.1% child life specialist, 11.1% grief support consultant) - Decedents’ age Fetal loss (36 weeks); 10 min old; 2 weeks old; and 7 months old	Semi-structured interviews	- The role of professional bereavement photography in perinatal loss was evaluated from the perspectives of parents, photographers and other professionals - Parents were asked to describe their experience of the photography session, namely its meaning, use, importance and impact on the bereavement process - Photographers shared their experiences of photographing parents - Health professionals discussed the benefits of this intervention for families	This study only included parents who had their children photographed when they were alive, unlike previous studies
Jones et al, USA, 2023^19^	Qualitative study	To understand the parents’ perceptions of LOI, as a reference for best practice in pediatric palliative care	-17 children’s family members (70.6% mothers, 11.8% fathers, 11.8% grandparents, 5.8% adult siblings) -Family members’ age (years): 18-39 (23.6%); 40-49 (17.6%); over 50 (58.8%) -Decedents’ age: from 6 months to 18 years	Semi-structured interviews	Bereaved parents were interviewed to explore their perceptions of “legacies”, to understand their definition, their manifestations and the factors that may influence the perception of these experiences	This article advocates personalized interventions centered on the meaning experienced by parents, not standard practices
Akard et al, USA, 2018^24^	Qualitative study	To investigate the significance of digital storytelling, as a legacy-making	-6 bereaved parents (50% mothers; mean age: 29.50 ± 6.35 years) -Decedents’ mean age: 1.67 ± 2.08 days	Semi-structured interviews	The bereaved parents discussed digital storytelling as a LOI in neonatal intensive care and provided suggestions on how to develop these interventions, particularly in terms of their acceptability, usefulness and their benefits	Participants in this study are parents who have lost a baby in a neonatal intensive care unit
Paul et al, USA, 2024^25^	Qualitative study	To study the significance of storytelling, as a legacy-making	- 19 bereaved parents (89.5% mothers) - Decedents’ age: from 1 to 24 years	Semi-structured interviews	This study explored the role of storytelling as a form of legacy, through the creation of narratives describing the (other) legacies left by their children, focusing on the impact they continue to have even after their children’s deaths	In addition to collecting data, these interviews ended up generating a product (the narrative) that also functioned as a legacy, highlighting this bi-directional link between narrative creation and legacy building
Walden et al, USA, 2021^26^	Qualitative study	To determine the significance of Heartbeat recording as a legacy-making	- 11 parents (100% mothers; aged 20-68 years) - Decedents’ sex: 54.5% male - Decedents’ age: from 9 days to 13 years	Semi-structured interviews	- A digital stethoscope was used to record the child’s heartbeat, which was then combined with a piece of music chosen by the parents to create a unique song - The parents then reflected on the benefits of this intervention as a legacy, in terms of its impact on bereavement, on their child’s experience of illness and on their coping mechanisms	This study involved parents of children with neurodegenerative diseases and promoted pre-loss care, focusing on the progressive loss of abilities and the associated progressive grief
Schaefer et al, USA, 2021^27^	Qualitative study	To understand the parents’ and children’s perceptions of LOI.	- 61 participants: 37 parents (70% mothers); and 24 children with advanced cancer (67% male) - Children mean age 14.04 ± 3.98 years	Semi-structured interviews	The interviews focused on how children with advanced cancer and their parents try to construct meaning in the face of the illness experience and the benefits of this intervention	- The children with advanced cancer also took part in this study - Children ≥8 years old provided self-report - Unlike other studies, this one focused on the construction of meaning before the child’s death
Hirsh et al, USA, 2023^28^	Qualitative study with surveys	To study the significance of videography, as a legacy-making for palliative care patients	- 9 children (aged ≥8 years)	Creation of a legacy video and the implementation of two surveys, one before and other after the video	- The intervention consisted of the creation of a legacy video in which children with life-threatening illnesses recorded personal messages, reflections or farewells - Before and after the recording, the participants completed questionnaires to assess their emotional state to understand the impact of the intervention	This study reflects the views of the children themselves as they go through their illness

Legend: LOI: legacy-oriented interventions; USA: united states of America.

**Table 2.** Main Results of the Individual Studies (n = 9)

Study, country, year	Benefits of legacy-oriented interventions	Impact on family grief
Barta et al, USA, 2024^11^	- Bond maintenance - Family engagement - Connection with healthcare team - Coping strategies - Meaning-making - Decreased anxiety and stress	Legacy building allows parents to come to terms with the death of their beloved children, but also to maintain bonds by perpetuating the child’s rituals and traditions. It is through this representation of the child that crucial tools are provided in the grieving process, as parents find these interventions meaningful even after the death of their children
Andrews et al, USA, 2020^12^	- Bond maintenance - Family engagement - Connection with the healthcare or the MTHS program teams	Parents who have been part of the MTHS programme report that this legacy has a lasting impact on the various stages of the grieving process, as it allows them to connect with the health care team that continues to support them after their child’s death
Ramirez et al, USA, 2019^13^	- Bond maintenance - Family engagement - Representation of the child - Meaning-making	Many benefits of photographs as a legacy intervention are reported these include finding meaning in these interventions; giving permission to grieve, as this is often not socially recognised; expressing different feelings and emotions; and helping to move through the distinct stages of grief
Jones et al, USA, 2023^19^	- Bond maintenance - Family engagement - Impact on others - Representation of the child - Coping strategies	Parents believe that legacies function as a way of representing characteristics and experiences and demonstrating the impact that has been left on other people. However, they emphasise that their perception of these interventions is dependent on continued support from health professionals and their own bereavement process, demonstrating a bidirectional relationship between grief and the creation of legacies
Akard et al, USA, 2018^24^	- Bond maintenance - Representation of the child - Coping strategies	Creating a legacy allows parents to maintain a bond with their deceased children. They can also introduce them to their relatives and, in the future, to other children who were too young to understand what had happened. In this way, parents say that this intervention helps them to come to terms with their grief
Paul et al, USA, 2024^25^	- Bond maintenance - Impact on others - Coping strategies - Meaning-making	In terms of creating a post-mortem legacy, parents emphasise the impact of their children on others and the acquisition of bereavement coping mechanisms that were influenced by the way their children dealt with their own illness
Walden et al, USA, 2021^26^	- Bond maintenance - Coping strategies - Meaning-making	Legacies make a positive contribution to grief, as they enable some parents to feel that they have a small piece of their children with them. These legacies allow parents to cope with their own bereavement and that of their family members
Schaefer et al, USA, 2021^27^	- Meaning-making - Benefit-finding	Both parents and young people with terminal illnesses argue that legacy-orientated interventions are a way of finding meaning. Difficulty in finding this meaning is associated with prolonged grief, so legacies play a key role in grieving process
Hirsh et al, USA, 2023^28^	- Symptom control - Coping strategies	The creation of a video by children with life-shortening conditions as a legacy intervention brings benefits to them. Particularly noteworthy is the reduction of anxiety and stress, facilitated by the existence of a safe space in which they can address various aspects related to the illness and death before it happens (anticipatory mourning)

Legend: MTHS: music therapy heart sounds; USA: united states of America.

**Table 3.** Risk of Bias in the Included Studies (n = 9)

Study; country; year	Total number of items	Number of “yes”	Number of “no”	Number of “unclear”	Number of “not applicable”	Jbi score	Degree of quality	Overall appraisal
Barta et al, USA, 2024^11^	10	8	0	2	0	0.8	High	Include
Andrews et al, USA, 2020^12^	10	7	1	2	0	0.7	Moderate	Include
Ramirez et al, USA, 2019^13^	10	8	0	2	0	0.8	High	Include
Jones et al, USA, 2023^19^	10	8	0	2	0	0.8	High	Include
Akard et al, USA, 2018^24^	10	8	0	2	0	0.8	High	Include
Paul et al, USA, 2024^25^	10	9	0	1	0	0.9	High	Include
Walden et al, USA, 2021^26^	10	8	0	2	0	0.8	High	Include
Schaefer et al, USA, 2021^27^	10	8	0	2	0	0.8	High	Include
Hirsh et al, USA, 2023^28^	10	8	0	2	0	0.8	High	Include

Extracted data were coded inductively by both authors. Codes were compared and refined through discussion until consensus was reached and then grouped into recurring themes. To facilitate synthesis and avoid redundancy, outcomes reported in the included studies were then organized into four broader thematic categories.

1. **Relational and memorialization outcomes:** Grieving process; Bond maintenance; Representation of the child; and Perceived impact on others
2. **Psychosocial adaptation outcomes**: Coping strategies; Meaning-making; and Benefit-finding
3. **Family and care system outcomes**: Family engagement; Connection with the healthcare team
4. **Clinical outcomes:** Symptom control

This thematic grouping provides a clearer synthesis of the available evidence and highlights both the breadth of LOI impact and areas where the evidence base remains limited.

## Results

### Study Selection

**Figure 1.**
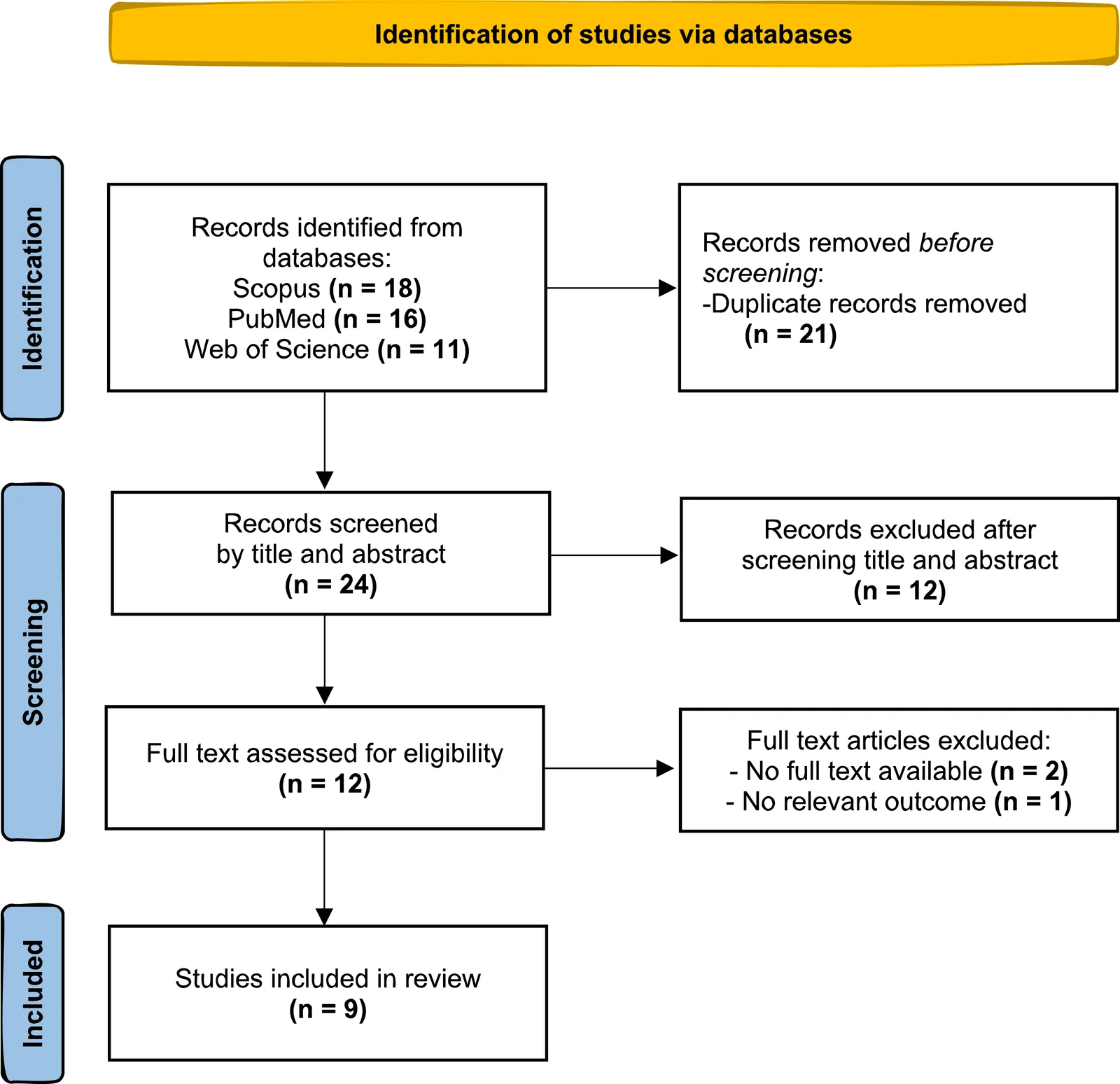
Flow Diagram of the Study Selection Process

The initial search yielded a total of 45 articles. After removing duplicates, 24 articles remained for screening. Of these, 12 were excluded based on title and abstract screening because they did not describe the intervention or outcome of interest. The remaining 12 articles underwent full-text analysis. Three were excluded at this stage: two due to the unavailability of the full text, and one because it did not meet the outcome criteria. Nine articles met the eligibility criteria and were included in this review. The selection process is summarized in the flow diagram (Figure 1).

### Study Characteristics

This review included nine qualitative studies, all conducted in the United States of America, involving a total of 164 participants.^11-13,19,24-28^

Most of the participants were bereaved parents or other family members.^11-13,19,24^ However, three studies collected data while the child was still receiving care in a hospital-based palliative care unit.^26-28^ In two of these studies, the children themselves were also interviewed.^27,28^

Some studies explored the nature of the interventions offered to children at the end of life and examined how legacy creation and meaning-making processes unfolded.^11,12,19,27^ Other studies focused on the development of specific interventions and their perceived impact on the child and family.^13,23,26-28^ Regardless of focus, all studies ultimately addressed the influence of these interventions on parents and their bereavement experience.^11-13,19,24-28^

The characteristics of the included studies are summarized in Table 1.

### Risk of Bias in Studies

The overall methodological quality of the studies was high, with only one study rated as moderate (Table 3). All nine studies met criteria for items 1, 2, 3, 4, 5, 8, and 10, consistently classified as “Yes.” These items evaluate the alignment between the philosophical perspective and research methodology (item 1); the appropriateness of the methodology for the research objectives (item 2); the coherence of data collection and analysis methods (items 3 and 4); participant representation (item 5); interpretation of results (item 8); and consistency between findings, implications, and conclusions (item 10). This consistency indicates a high level of methodological rigor across studies.

However, common limitations emerged in items 6 and 7, which concern reflexivity. Item 6 assesses whether researchers’ beliefs, values, and theoretical-cultural positioning were made explicit. All studies were rated “Unclear” on this item, as none included formal statements or reflexive critiques addressing researcher positioning.

Item 7, which examines whether the potential influence of the researcher on data generation and interpretation was considered, was also mostly rated “Unclear.” The exception was the study by Paul et al,^25^ which explicitly stated that no prior relationship existed between interviewers and participants, warranting a “Yes” classification.

Regarding ethical considerations (item 9), eight of the nine studies clearly reported obtaining ethics committee approval and were rated “Yes.” Only Andrews et al^12^ lacked an explicit ethics statement and was therefore rated “No” for this item.

### Results of Individual Studies

The main findings of the included studies are summarized in Table 2, where the benefits of LOI are outlined in relation to the study outcomes, and its impact on family grief is highlighted.

### Results of Synthesis

The included studies reported outcomes across the four thematic categories, though not all categories were represented consistently. Relational and memorialization outcomes were the most frequently described, reflecting LOI’ roles in sustaining bonds and creating enduring memories. Psychosocial adaptation outcomes were also well supported, highlighting LOI’ contributions to resilience and emotional adjustment. In contrast, family and care system outcomes appeared less frequently, and evidence for clinical outcomes was scarce. This distribution indicates that while LOI are primarily oriented toward relational and psychosocial domains, their broader potential within family and clinical contexts remains underexplored. This variation underscores both the areas in which LOI have demonstrated impact and the significant gaps that remain for future research.

## Relational and Memorialization Outcomes

### Grieving Process

Grief was reported in all of the included studies.^11-13,19,24-28^ Many studies suggest that the grieving process can be positively influenced by the outcomes of LOI, particularly through maintaining emotional ties with deceased children.^11,24^ Moderate-quality evidence showed that these legacies foster a sense of closeness to the healthcare team, which has been shown to be beneficial, as the team continues to offer support in the grieving process even after the child’s death.^12^ The impact of these legacies also varies depending on the parents’ stage of grief.^19^ The objects created through LOI allow the child to be introduced to present and future family members, which has been found to ease parents’ grief.^24^ Additionally, LOI offer parents the opportunity to express feelings and emotions at different stages of the grieving process, facilitating emotional release.^13^ Legacies help represent the child in various aspects, such as their characteristics, beliefs, coping strategies, and values.^11,26^ The ability to adopt effective coping mechanisms during bereavement often depends on these personal characteristics of the child.^25^ Another key factor influencing family bereavement is the ability to find meaning and purpose, as the lack of such meaning can contribute to a prolonged grieving process.^27^

### Bond Maintenance

Bond maintenance was discussed in seven articles.^11-13,19,24-26^ Developing legacy-oriented activities enables parents to maintain a connection with their children after their death.^11-13,19,24-26^ For tangible legacies, this connection is preserved through the creation of objects that serve as physical representations of the child.^11,13,19,24,26^ For intangible legacies, the maintenance of bonds is rooted in the preservation of the child’s rituals or experiences.^11,13,19,25^

### Representation of the Child

Representation of the child was discussed in three studies.^13,19,24^ In some cases, particularly in neonatal intensive care units where parents may have limited time with their children, a visual representation can serve as a powerful form of validation.^13,24^ A visual representation not only preserves the child’s memory but also fosters a sense of closeness to the deceased child.^13,24^ Given the brief life span of some infants, such representations also provide a way for family members who did not have the opportunity to meet the child to form a connection.^13,24^ Additionally, these representations offer future family members a chance to get to know the child.^13^

The physical representation of a child (eg, photographs) provides numerous benefits, such as facilitating difficult conversations, allowing the sharing of stories and memories, and helping to alleviate the fear of forgetting.^13^ In an intangible sense, each legacy serves as a symbol of the child’s unique characteristics.^19^

### Perceived Impact on Others

Two studies reported on the impact of LOI on others.^19,25^ Paul et al^25^ highlight that bereaved parents place significant importance on the impact their children had on other people. Furthermore, it is crucial for parents that this impact continues to be recognized by others after their child’s death, helping to perpetuate the child’s memory.^25^ Jones et al^19^ emphasize that the legacy of these children is reflected in others through changes in attitudes and behaviours, including an increase in altruism, respect, and inclusion. This impact is also seen in the organization of charity events, honor walks, and the preservation of the child’s rituals.^19^

## Psychosocial Adaptation Outcomes

### Coping Strategies

Six of the studies examined coping strategies as a benefit of LOI.^11,19,24-26,28^ LOI are specifically designed to help families cope with challenging health experiences.^19,24,26^ To enhance their acceptability, these interventions must align with each family’s values and beliefs.^11,19^ Coping strategies identified in the studies include maintaining bonds, making meaning, and reflecting on the child’s experience of illness.^19,24-26^ Additionally, spirituality,^19,26,28^ and humour,^19,26^ were also recognized as adaptive coping mechanisms.

### Meaning-Making

The creation of meaning was identified as an outcome in five studies.^11,13,25-27^ Barta et al^11^ argue that the legacies created enable parents to find meaning and purpose in their child’s illness. Walden et al^26^ further suggest that meaning-making may arise from the validation of the child’s life. After the child’s death, these legacies remain significant for families, as they provide a way to honor the child’s memory, thus continuing the process of meaning creation.^25^ It is important to note that the meanings attributed to these legacies are not static; they evolve over time in relation to the stages of bereavement that families experience.^13,26^ For parents, accepting a child’s illness can be a profound challenge, often leading them to interpret the illness through religious, spiritual, scientific, or environmental lenses.^27^ Additionally, sharing the history and legacy of their children is another way parents find meaning.^27^

### Benefit-Finding

One study explored the benefits of creating a legacy.^27^ Schaefer et al^27^ found that for parents, the experience of their children’s illness led to a shift in their outlook on life, helping them focus on what truly mattered and fostering increased positivity and hope. Parents also reported that the illness strengthened family relationships, friendships, and spirituality.^27^

## Family and Care System Outcomes

### Family Engagement

Family engagement was reported in four studies.^11-13,19^ Barta et al^11^ and Jones et al^19^ emphasize that LOI should be personalized and tailored to the unique characteristics and values of each child and their family. This alignment between the intervention and the family’s principles fosters a sense of being heard and involved in the entire process.^11,12^ The active participation of families in the creation of legacies enhances their acceptance and compliance with LOI.^11^

From a different perspective, in the context of neonatal loss, legacies provide physical evidence, such as photographs, which validate the existence of the baby and reaffirm the parents’ roles, helping to preserve the memory and significance of the child.^13^

### Connection with the Healthcare Team

This subject was addressed in two studies.^11,12^ Healthcare professionals play a critical role in providing support tools for parents and families, addressing various aspects such as illness management, the creation of legacies and meaning, and the grieving process.^11^ Barta et al^11^ further emphasize that the best practices of healthcare professionals are significantly shaped by feedback from bereaved parents, who, after experiencing the full process of illness and loss, offer valuable insights and recommendations that help improve care. This highlights the collaborative, two-way relationship between parents and healthcare professionals.^11^ Andrews et al^12^ suggest that, although the creation of legacies involves multiple healthcare professionals, these interventions are best introduced by bedside nurses, given their closer proximity to patients and the deeper bonds they develop over time. However, generalizing these results may be limited due to the moderate risk of bias in the study.^12^

## Clinical Outcomes

### Symptom Control

One study specifically focused on symptom control.^28^ LOI were found to provide a safe space for children to express their feelings, thoughts, and fears, which helped alleviate anxiety related to both the illness and the prospect of death.^28^

## Discussion

### Summary of Evidence

This systematic review synthesized evidence on LOI in PPC and its impact on the bereavement process of families following the loss of a child. The primary aim was to assess the effectiveness of these interventions in supporting family grief, maintaining bonds with the deceased child, and promoting psychosocial adaptation during the mourning period. Nine qualitative studies, conducted exclusively in the United States of America, involving a total of 164 participants, were included in the review. The studies focused on bereaved parents and family members, with some also exploring the perspectives of children receiving palliative care.

The evidence showed that LOI focus on creating lasting memories of the child, through tangible or intangible legacies, which can support families through grief and help maintain connections after the child’s death. The key themes found in the studies were.

• **Grieving Process:** LOI help parents express emotions, maintain connections, and find comfort, easing the grieving journey.
• **Bond Maintenance**: Legacies allow enduring ties with the deceased child, whether through physical mementos or preserved traditions.
• **Representation of the Child**: LOI validate parental experiences, preserve the child’s memory, and symbolically represent their unique traits, helping to alleviate the fear of forgetting.
• **Perceived impact on Others**: Legacies resonate beyond the immediate family, inspiring community remembrance, shifts in attitudes, and acts of charity.
• **Coping Strategies**: Families use LOI to manage emotional distress, drawing on spirituality, humor, and shared experiences aligned with their values.
• **Meaning-Making**: Creating legacies helps families find purpose in their loss, with meanings evolving over time and across stages of grief.
• **Benefit-Finding**: Legacy interventions helped parents reframe their experiences, fostering hope, deeper relationships, and strengthened spirituality.
• **Family Engagement**: Personalized legacy interventions promote active family involvement, reinforcing parental roles and affirming the significance of the child’s life.
• **Connection with the Healthcare Team**: Clinicians, especially nurses, play a pivotal role in facilitating LOI and maintaining supportive relationships.
• **Symptom Control:** LOI may reduce anxiety in children by offering a safe outlet for emotions and fears related to illness and dying.

## Relational and Memorialization Outcomes

### Grieving Process

There is evidence that the death of a child is a tragic and traumatic experience for parents^14,20,29,30^ that can affect the whole family system.^20^ Parental grief following the loss of a child is considered one of the most intense and painful forms of bereavement^14,29^ with psychological,^14,30^ social and health consequences.^14^ These parents are therefore at increased risk of developing prolonged grief processes.^29,30^ Parents who received these interventions had fewer symptoms of prolonged grief, demonstrating a positive effect on the bereavement process.^31-33^

### Bond Maintenance

In our review, all articles mentioned the maintenance of bonds as a benefit of the LOI. Consistent with this, other studies have shown that this bond is important for parents to be able to cope with bereavement.^13,32^ However, there are studies that show that the continuation of bonds may contribute to maladaptive bereavement processes, as these depend on different attachment styles.^15^ While some people benefit from this connection, others with insecure attachment styles may need to distance themselves from the deceased and readjust their lifestyle.^15^

### Representation of the Child

In the literature there are different approaches to legacies and how they represent children. On the one hand, this representation can take place through the creation of tangible legacies that perpetuate the characteristics of children.^10,32^ On the other hand, it can be represented symbolically, as a complex representation of their relationships, experiences and interactions.^5,6^ In either case, both representations allow parents to keep the memory of their children alive after their death.^6,10^

### Perceived Impact on Others

The legacies can change other people’s perspectives and ways of thinking, and it is perceived as having a positive and lasting effect on others.^5,15^

## Psychosocial Adaptation Outcomes

### Coping Strategies

LOI promotes important adaptative coping mechanisms during illness and later bereavement and provides tools for managing illness-related stressors.^32^ Recent studies have also tried to understand the potential of LOI to provide coping mechanisms for sick children.^33^ Coping showed a slight increase in the intervention group, but the change was not statistically significant.^33^

### Meaning-Making

The literature also describes the utility of LOI for meaning-making.^10,32^ LOI enables families to make sense of their experiences and integrate their loss.^5^ On the other hand, it’s been found that difficulty in making sense of or coming to terms with someone’s death is associated with a greater likelihood of experiencing maladaptive grief.^16,17^

### Benefit Finding

According to literature, parents try to make their children’s lives known and meaningful, to prolong their legacies.^5^ So they strive to keeping their children’s memories alive.^18^

Parents try to help other families who are going through similar situations of illness or loss.^5^ These attitudes are also beneficial for the bereaved parent’s own grief.^18^

## Family and Care System Outcomes

### Family Engagement

LOI promote the development of patient-and-family-centered activities.^7^ It allows the bereaved to create memories, validate the children’s lives and perpetuate their family unity.^5^ These creations provide families with a sense of relief from the many stressors associated with illness and hospitalisation.^7^

Legacies are seen by many parents as something meaningful and important.^32^ Controversially, there have also been cases of family members experiencing feelings of guilt, regret, loss and sadness during the development of these projects or after child’s death.^32^

### Connection with the Healthcare Team

As our study has shown, the connection with the healthcare team has positive outcomes in the bereavement process, due to the support provided before and after the death.^7,14,30^

## Clinical Outcomes

### Symptom Control

Concordantly, the literature suggests that these interventions tend to promote a reduction in anxiety and sadness.^5,32^ Caregivers also experienced symptom control, particularly with a reduction in anxiety.^8^ However, some authors emphasize that there is no significant difference when compared to those who did not receive these interventions.^32^

## Strengths: Relevance of the Legacy-Oriented Interventions to Key Groups

### Families

The findings underscore the importance of offering bereaved families opportunities to maintain connections with their deceased child, providing a sense of meaning, and fostering adaptive coping mechanisms during the grieving process.

### Healthcare Providers

This review highlights the importance of integrating LOI into PPC practices. Training healthcare professionals, particularly nurses, in facilitating these interventions could significantly enhance the support provided to families.

### Policy Makers and Practitioners

Understanding the potential benefits of LOI can inform policy development regarding palliative care services for children and families, emphasizing the need for holistic, personalized care that addresses both physical and psychosocial aspects of grief.

In conclusion, while the evidence indicates that LOI have the potential to support bereaved families and aid in the grieving process, further research is needed to explore their long-term impact and the ways in which these interventions can be optimized and implemented in various healthcare settings.

## Limitations

This systematic review has several limitations that should be acknowledged.

First, although a comprehensive search strategy was used across three major databases, relevant studies may have been missed due to language restrictions, database selection, unpublished literature, or “grey literature” not being included.

Second, the review was limited to qualitative studies conducted in the United States of America, which may reduce the generalizability of the findings to other cultural or healthcare contexts.

Third, the synthesis was narrative in nature, as the heterogeneity of interventions, outcomes, and study designs precluded meta-analysis or more structured comparison. Additionally, no quality threshold was applied to exclude lower-quality studies; instead, all eligible studies were included and critically appraised to reflect the breadth of available evidence.

Fourth, because of the heterogeneity of study designs, populations, and outcome measures, not all thematic categories could be assessed across all included studies, which limits the comparability and generalizability of findings.

Lastly, the reliance on subjective interpretations from qualitative data introduces the possibility of interpretive bias, despite efforts to ensure rigor through independent screening and consensus-based synthesis.

## Conclusions

This review highlights the meaningful role of LOI in supporting the bereavement process of families following the loss of a child. The findings suggest that LOI help maintain emotional bonds, promote adaptive coping, and foster meaning-making, contributing to healthier psychosocial adaptation during grief. The interventions also enhance family engagement and communication with healthcare professionals, with some evidence indicating benefits for symptom control in children and positive ripple effects on others.

Although the available evidence is qualitative and limited to studies conducted in the United States of America, it provides important insights into how LOI can be integrated into PPC to meet the emotional and existential needs of both children and their families. These findings support the relevance of LOI as family-centered care strategies that extend beyond the child’s death.

Future research should explore LOI in more diverse cultural and healthcare settings, examine long-term outcomes, and develop standardized approaches to guide implementation in clinical practice. Involving bereaved families in the co-design of interventions may also help ensure that LOI are meaningful, respectful, and impactful.

## Data Availability

All data relevant to this study are included within the article. No additional datasets, code, or materials were generated or used.*

## Appendix

### List of Abbreviations and Acronyms

LOI: Legacy-Oriented Interventions
PPC: Pediatric Palliative Care.
